# Preserved fascicular architecture predicts neuroma pain: a morphometric study

**DOI:** 10.1186/s40478-025-02154-1

**Published:** 2025-12-01

**Authors:** Luis A. Pardo, Carolina Thomas, Arndt F. Schilling, Christine Stadelmann, Jennifer Ernst

**Affiliations:** 1 Hannover Medical School, Department of Trauma Surgery, Hannover, Germany; 2 Department of Trauma Surgery, Orthopaedics and Plastic Surgery, University Medical Center Göttingen, Göttingen, Germany; 3 Department of Neuropathology, University Medical Center Göttingen, Göttingen, Germany; 4 Paul Flechsig Institute – Centre of Neuropathology and Brain Research, Medical Faculty, Leipzig University, Leipzig, Germany

**Keywords:** Neuroma pain, Peripheral nerve injury, Neuropathic pain, Nerve morphology

## Abstract

Painful neuromas remain a major clinical challenge after limb amputation and peripheral nerve trauma. While histological features such as inflammation, fibrosis, and axonal sprouting have been proposed as contributors to neuropathic pain, direct clinicopathological correlations remain inconsistent. The role of internal nerve architecture, particularly the proportion of preserved, organized fascicular tissue, has not been quantitatively assessed in relation to pain intensity. To address this gap, this study investigates whether the relative amount of organized versus unorganized nervous tissue within neuromas correlates with patient-reported pain, independent of classical histological parameters. Accordingly, we performed whole-slide histological segmentation of peripheral nerve samples including control nerves, non-painful neuromas, and painful neuromas. Tissue compartments, including organized fascicles, unorganized neuroma tissue, connective tissue, and adipose tissue, were quantified and correlated with clinical pain scores. Our results demonstrate that painful neuromas exhibited a significantly lower relative amount of organized nervous tissue compared to non-painful neuromas (*p* = 0.006), while total nerve size and other tissue components showed no significant differences. A strong negative correlation was observed between pain intensity and the relative amount of organized fascicular tissue (r = − 0.82, *p* < 0.001). No correlation was found between pain and the absolute amount of unorganized nervous tissue or connective tissue. Taken together, these findings suggest that the structural preservation of organized nerve fascicles modulates the clinical expression of neuroma-related pain. Morphometric assessment of fascicular organization may provide a new biomarker for surgical planning and outcome prediction in neuroma management.

**Supplementary Information:**

The online version contains supplementary material available at 10.1186/s40478-025-02154-1.

## Introduction

Chronic neuropathic pain remains one of the most debilitating sequelae of limb amputation and major peripheral-nerve trauma. Up to 50% of amputees report persistent residual-limb pain attributed to a neuroma at the transected nerve stump [1], yet not every neuroma is painful, and the reasons for this discrepancy are still poorly understood [2, 3]. Proposed mechanisms range from excessive connective-tissue scarring and aberrant axonal sprouting to prolonged neuro-inflammation, but direct clinicopathological correlations are rare.

Neuroma formation follows failed target organ re-innervation after nerve injury. When regenerating axons are unable to re-enter their original endoneurial tubes, they form a disorganized mass containing so called mini fascicles surrounded by connective tissue and infiltrating immune cells at the proximal stump [4, 5]. Histologically, this structure is often described as unorganized and fibrotic nervous tissue [6, 7], but it remains unclear which morphological or cytoarchitectural features, if any, predict pain severity.

Most studies to date have focused on potential generators of aberrant nociceptive signaling, including inflammatory cell infiltration [8], myofibroblast activity [9, 10], sodium channel accumulation [11, 12], and local mechanical compression [2]. However, the results have been inconsistent. For example, shifting macrophages toward an M1 pro-inflammatory phenotype in rodents does not reliably increase pain behavior [13]. More recent transcriptomic analyses have identified specific immune cell subsets, such as MARCO⁺ macrophages, that correlated with pain at the RNA level, but these findings have not been confirmed histologically [14].

Fibroblast-related mechanical stress has been implicated in neuroma-associated pain. A strong correlation was observed between α-smooth muscle actin (α-SMA) expression and pain intensity in human neuromas, suggesting a role for myofibroblast contraction [9]. This has been supported in a rat model, where modulation of α-SMA levels influenced autotomy behavior, though with variable outcomes [10]. However, α-SMA’s expression is part of a broader tissue response involving nerve growth factor (NGF) production [15], vascular remodeling [16], and hypoxia [17] signaling-processes essential for wound healing but not specific to pain transmission. Thus, α-SMA may reflect ongoing tissue repair rather than directly causing pain, underscoring the multifactorial nature of neuroma-associated pain and the need to look beyond fibrotic markers alone [3].

Meanwhile, studies examining gross morphometric markers such as cross-sectional area or the neuroma-to-nerve diameter ratio have shown no predictive value for clinical pain intensity [1, 18, 19]. Despite decades of research into why neuromas hurt and where the aberrant signal comes from, little is known about how the internal architecture of the nerve itself might modulate the perception of pain.

In this study we propose that the degree of preserved, organized fascicular tissue within a neuroma may act as a structural buffer against neuropathic pain, regardless of the specific source of nociceptive input. To test this hypothesis, we applied a machine learning–assisted morphometric analysis to histological cross-sections of control nerves, painless neuromas, and painful neuromas. Our goal was to quantify the proportions of healthy (or organized) nervous tissue, unorganized nervous tissue, fat, and collagen and to evaluate their relationship to patient-reported pain levels.

## Materials and methods

### Cohort and human tissue collection

Nerve samples of 12 patients were obtained during residual limb revision surgery and sarcoma surgery. Approval was obtained with a waiver of informed consent, per routine standard of care. The samples obtained were classified in three groups (see Supp. Table 1), obtained from three groups of patients.

*Painful neuromas*: This group consisted of upper and lower limb amputees with residual limb pain clinically attributable to a neuroma. Painful neuromas were defined by the presence of a positive Hoffmann–Tinel sign [20] at the residual limb stump, temporary pain relief after local anesthetic infiltration, and characteristic imaging findings on MRI or ultrasound. (Median age 32 years, range 23–61; 4 male, 1 female; median NRS 7, range 5–8).

*Non-painful neuromas*: This group included transected, bulbous proximal nerve ends identified intraoperatively during revision surgery for other residual limb pathologies (e.g., scars, soft-tissue problems, socket-related issues). These neuromas showed no clinical signs of pain: the Hoffmann–Tinel sign was absent, and NRS was consistently 0. MRI/ultrasound was not systematically performed in this group. (Median age 40 years, range 38–55; all male; NRS consistently 0).

*Controls*: The third group consisted of normal nerve samples harvested during tumor resections (median age 56 years, range 25–77; 2 male, 2 female; NRS 0).

All neuromas in groups 1 and 2 originated from Sunderland grade V injuries (complete transections, either from amputation or tumor resection). If present, such nerve ends were resected and transposed surgically like painful neuromas [21, 22].

### Histology and imaging

Upon surgical collection, samples were transported to neuropathology, on a wet cotton gauze devoid of fixatives for clinical standard neuropathological examination. After macroscopic inspection by a trained neuropathologist, the tissue was fixed in 3.7% formalin solution for 12–24 h and embedded in paraffin. 5–6 µm-thick paraffin sections were cut and histological stainings were performed. For histological analyses, besides H&E, Elastica van Gieson (EvG) staining was applied to assess the overall tissue architecture and the composition of connective tissue fibers. Whole-slide images were acquired at 200 × using a VS120 virtual slide microscope (Olympus) and the cellSense Dimension software (Olympus). Images were stored and annotated using an OMERO server [23].

### Machine learning-based whole slide image analysis

**Fig. 1:**
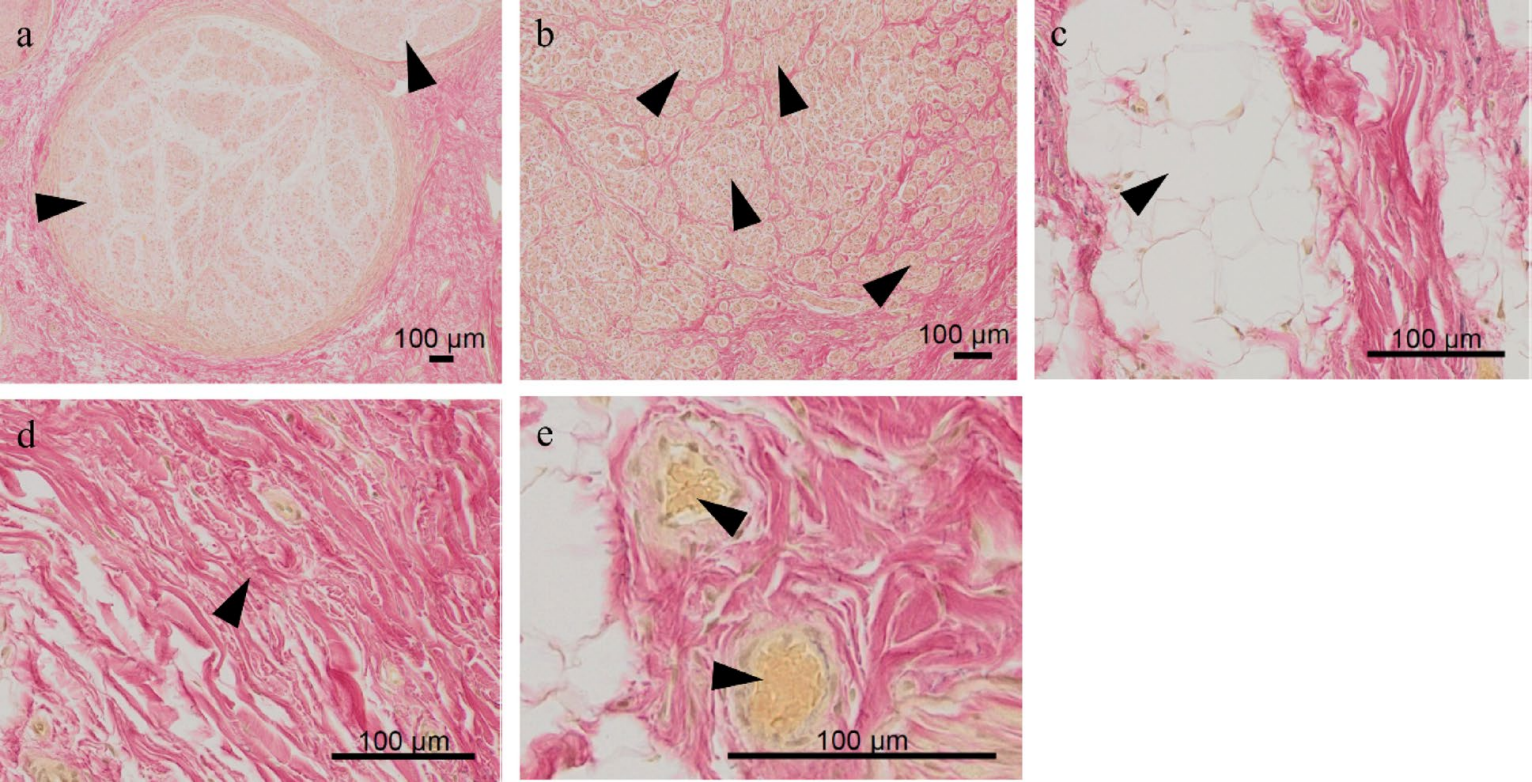
Tissue qualities. Microscopic images of various tissue types with a 100 μm scale bar. **a** Organized nervous tissue, **b** unorganized nervous tissue, **c** adipose tissue, **d** connective tissue, and **e** erythrocytes. Black arrows indicate the specific tissues within each image

In order to segment and quantify the tissue components of the sample (including organized and disorganized nervous tissue) EvG whole slide images were analyzed using a random forest classifier from scikit-learn [24]. Tissue components were annotated using regions of interest (ROIs) in each image, classified into categories such as “organized nervous tissue” (healthy nerve fascicles) and “unorganized nervous tissue” (neuroma), connective tissue, adipose tissue, and erythrocytes (Fig. 1).

These ROIs were transformed into masks and used to train a random-forest classifier. The classifier predicted tissue types in the images, outputting a 6-dimensional matrix. Post-processing included filtering based on fascicle roundness to distinguish healthy fascicles from neuromas. Finally, the masks were analyzed to calculate the relative areas of each tissue type and the ratio of healthy to unorganized fascicles using the following formula $\frac{unorganized-organized}{\mathrm{max}(unorganized, organized)}$. This results in a “normalized deviation index” between -1 and 1, from which 1 means, that only unorganized nervous tissue is present and -1, that only organized nervous tissue is present (which is only true for the control group) (detail description in Supplementary Material 1).

### Statistical analysis

Kolmogorov–Smirnov tests of normality were used to verify the data´s normal distribution. To compare the relative amounts of tissue between the groups, we performed t-tests for independent samples between both populations. As in the case of the relative and absolute amount of unhealthy fascicles in controls the results cannot possibly be normal distributed, we performed Mann–Whitney U tests on these cases. As the number of samples is small, we calculated Spearman correlation coefficients with associated p-values between the different tissue-percentages and ratios and pain levels reported by the patients. All results are presented as “median [interquartile range (IQR)]”. Plots and statistical annotations were generated in Python using the Seaborn [25] and Statannotations [26] libraries.

## Results

**Fig. 2:**
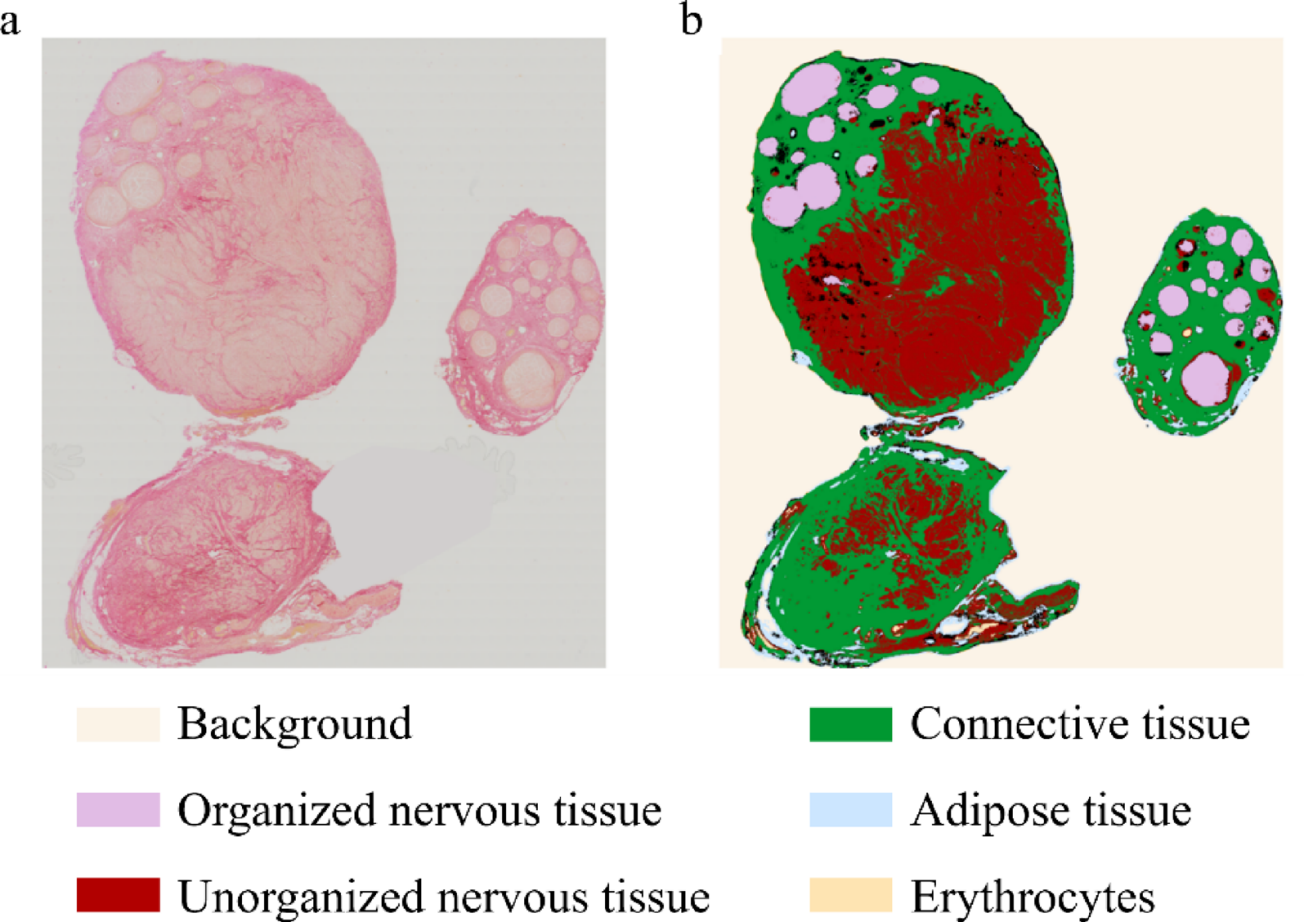
Segmented nerve. **a** Raw whole-slide image (EvG staining) and **b** segmented image as output from the algorithm (exemplary for one patient)

To assess whether the structural composition of neuromas is associated with neuropathic pain, we performed a morphometric analysis comparing the relative and absolute amounts of organized nervous tissue, disorganized nervous tissue, adipose tissue, collagen, and erythrocytes in healthy nerves and neuromas from patients with varying degrees of neuropathic pain utilizing a random forest classifier (Fig. 2).

### Healthy nerves vs. Neuromas: Neuroma patients display an increased amount of unorganized nervous tissue and a decreased relative amount of adipose tissue compared to controls.

**Fig. 3:**
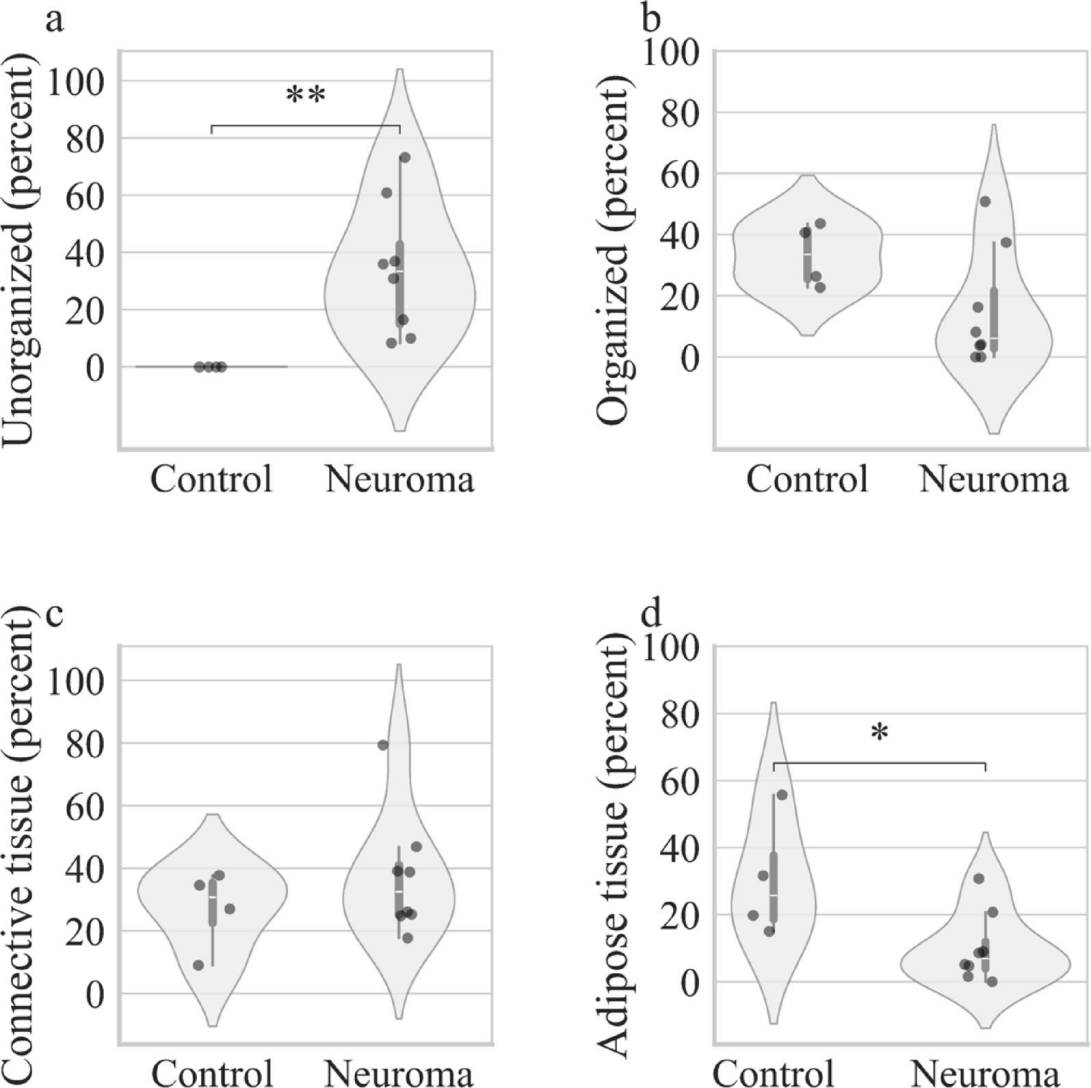
Healthy nerves vs. Neuromas: Relative amount of unorganized, organized, connective and adipose tissue **A** Relative area (percent of total tissue area per sample) occupied by unorganized nervous tissue, **B** organized nervous tissue, **C** connective tissue, and **D** adipose tissue in peripheral nerve control samples and neuromas. Each violin plot represents the density of data points at different values. The white dot indicates the median, and the thick bar represents the interquartile range (IQR). An asterisk (*) indicates *p* < 0.05, two asterisks (**) indicate *p* < 0.01

In the comparison between control nerves and neuromas, we found a significantly higher relative amount of unorganized nervous tissue in neuromas (p = 0.003), as well as a significantly lower proportion of adipose tissue (p = 0.01). No significant differences were detected for the relative amounts of organized nervous tissue or connective tissue (Fig. 3).

**Fig. 4:**
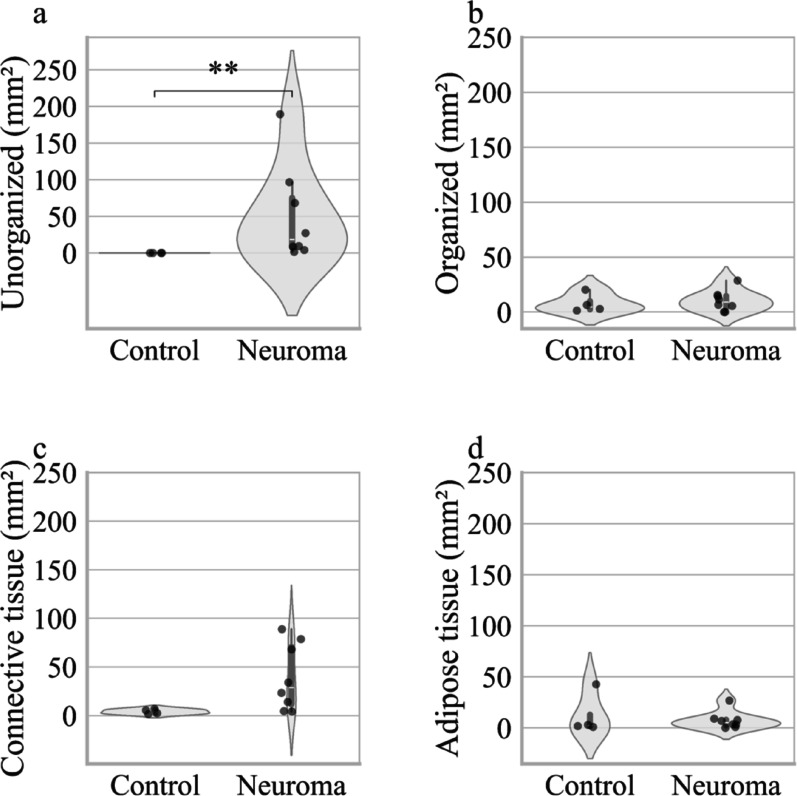
Healthy nerves vs. Neuromas: Absolute Amount of unorganized, organized, connective and adipose tissue **a** Absolute amount of unorganized nervous tissue, **b** organized nervous tissue, **c** connective tissue, and **d** adipose tissue in peripheral nerve control samples and neuromas. Each violin plot represents the density of data points at different values. The white dot indicates the median, and the thick bar represents the interquartile range (IQR). An asterisk (*) indicates p < 0.05, two asterisks (**) indicate *p* < 0.01

Comparing control nerves and neuromas in terms of absolute tissue area covered by the different tissue qualities, we again found a significantly increased amount of unorganized nervous tissue, but no difference in any other tissue quality (Fig. 4).

### Painful vs. non-painful neuromas: painful neuromas have significantly less organized nervous tissue

**Fig. 5:**
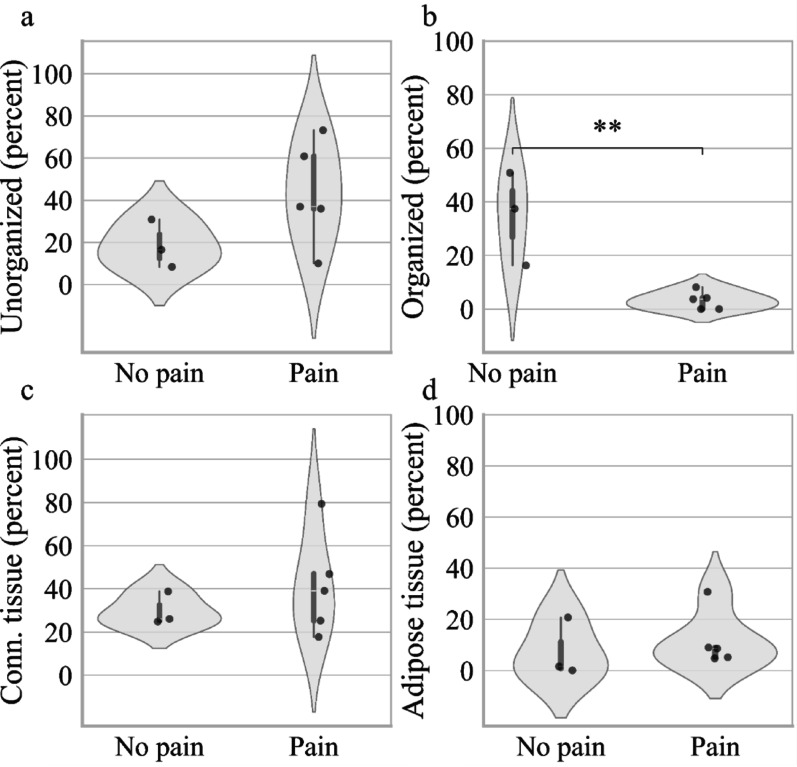
Painful vs. Non-painful Neuromas: Relative Amount of unorganized, organized, connective and adipose tissue **a** Relative area (percent of total tissue area per sample) occupied by unorganized nervous tissue, **b** organized nervous tissue, and **c** connective tissue, and **d** adipose tissue of painful neuromas and neuromas. Each violin plot represents the density of data points at different values. The white dot indicates the median, and the thick bar represents the interquartile range (IQR). Two asterisks (**) indicate p < 0.01

**Fig. 6:**
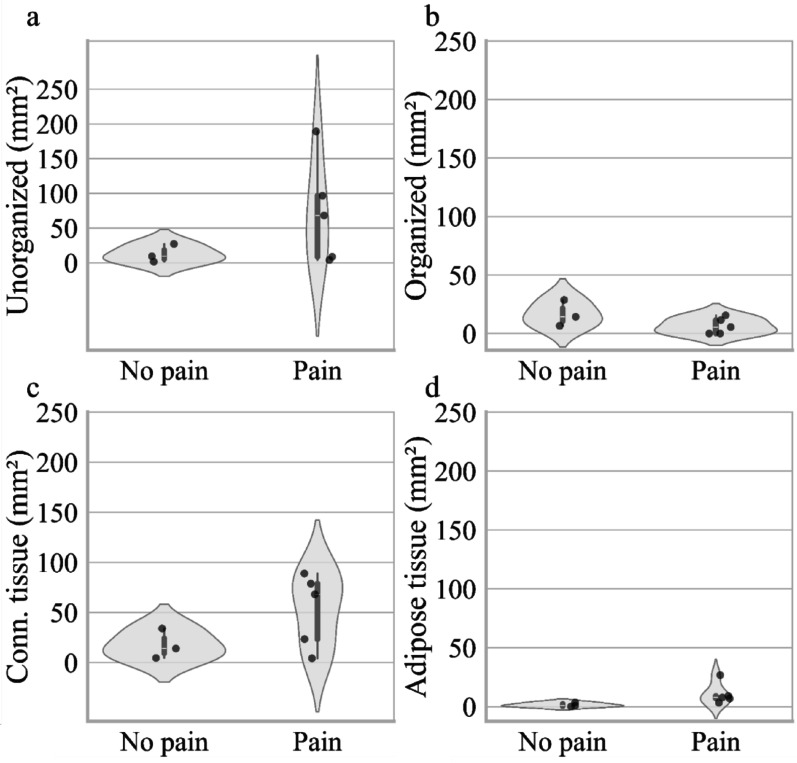
Painful vs. Non-painful Neuromas: Absolute Amount of unorganized, organized, connective and adipose tissue **a** Absolute amount of unorganized nervous tissue, **b** organized nervous tissue, and **c** connective tissue, and **d** adipose tissue of painful neuromas and neuromas. Each violin plot represents the density of data points at different values. The white dot indicates the median, and the thick bar represents the interquartile range (IQR)

Within the neuroma subgroup, the relative amount of organized nervous tissue was significantly lower in painful neuromas (p = 0.006, Fig. 5), although the absolute area of this tissue component was not significantly different (Fig. 6). Other parameters, including the relative and absolute amounts of unorganized nervous tissue, connective tissue, adipose tissue showed no significant differences between painful and non-painful neuromas (Figs. 5 and 6).

### Correlation with pain: preserved organized nervous tissue negatively correlates with neuroma pain

**Fig. 7:**
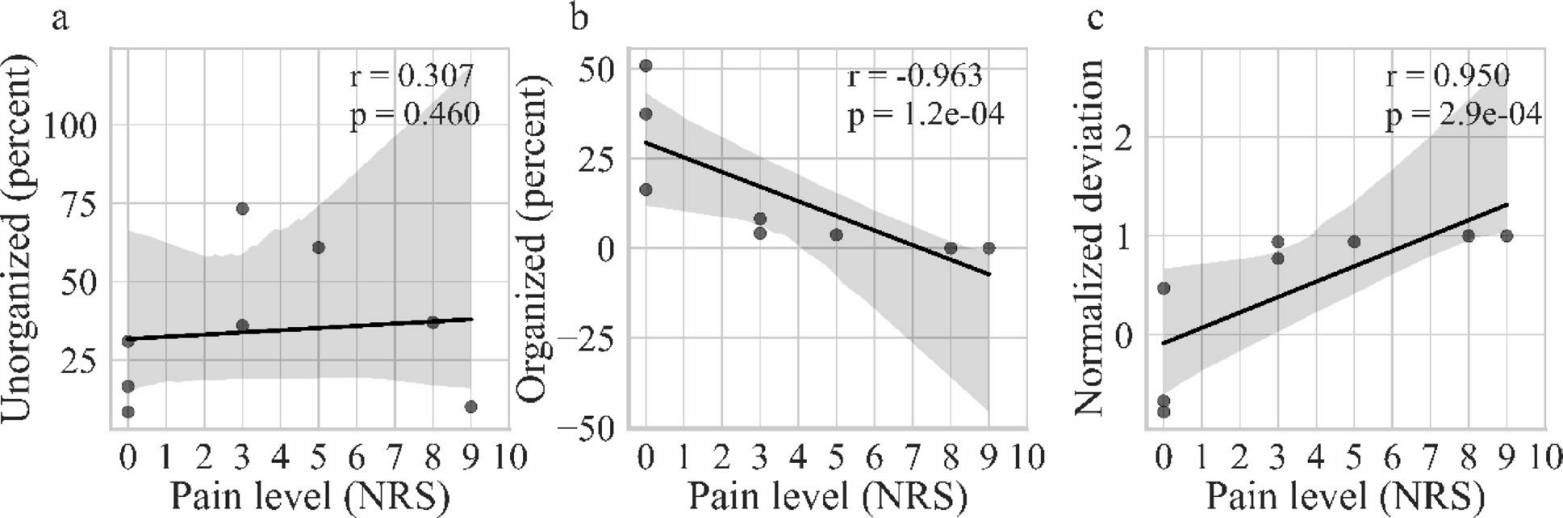
Correlation of analyzed tissue quality vs. pain level (NRS). Correlation between **a** the relative amount of unorganized nervous tissue and **b** organized nervous tissue, and **c** the normalized deviation between organized and unorganized nervous tissue in neuromas in correlation to the pain level (VAS) reported by the patients. The correlation coefficient (r) and p-value (p) were calculated using Spearman’s correlation

We found no correlation between pain and the relative amount of unorganized nervous tissue. However, a significant negative correlation between the relative amount of organized nervous tissue and pain intensity (measured via NRS; *p* < 0.001), and a positive correlation between the normalized deviation index and pain (*p* < 0.001) was detected (Fig. 7). Exploratory analyses did not reveal significant associations of age (r = − 0.20, *p* = 0.64) or sex (*p* = 0.18) with reported pain intensity.

## Discussion

We demonstrate that a lower relative amount of organized nervous tissue within neuromas is significantly associated with increased pain intensity, suggesting that structural preservation plays a key protective role, regardless of the underlying source of nociceptive input.

While prior studies have focused on inflammatory infiltrates or mechanical stressors like connective tissue proliferation as primary drivers of neuroma pain [9, 11, 12, 27, 28], our findings shift attention toward the internal nerve architecture itself. Although connective tissue was present in both neuromas and control nerves, we observed no significant differences in its relative or absolute amounts between groups. This supports previous findings that while connective tissue is commonly seen in neuromas, its abundance is not necessarily pathognomonic [29]. Notably, our segmentation approach did not differentiate between intrafascicular and extrafascicular connective tissue neither distinguished between perineurial cells and fibroblasts, which could influence its pathological relevance. Moreover, connective tissue proliferation may be influenced by prior surgical interventions [30], possibly explaining inconsistencies across studies. In our cohort, however, connective tissue quantity showed no correlation with reported pain intensity, suggesting that its presence and implication in the generation of aberrant signaling alone is insufficient to account for inter-individual differences in neuroma pain.

Further, our findings highlight significant disparities in the relative amount of adipose tissue between control nerves and those affected by neuromas, challenging the traditional view that does not consider adipose tissue a key factor in neuroma-related pain. This observation prompts consideration of lipid metabolism’s impact on nerve injury and repair processes. Research indicates elevated levels of specific lipids, like phosphatidylcholine, sphingomyelin, and ceramides, in injured nerve tissues, with ceramide levels notably correlating with the severity of diabetic neuropathy [31–33]. Yet, it remains uncertain whether these changes in lipid metabolism are unique to neuromas or represent a universal response to (peripheral) nerve damage. A comparative analysis revealed no significant difference in the absolute amount of adipose tissue between the control and neuroma groups, suggesting that the total adipose tissue content remains unchanged. However, neuroma growth appears to reduce the proportion of other tissues relative to adipose tissue. Beyond serving as energy storage, adipose tissue may offer protective benefits, as evidenced by the pioneering work of Millesi et al., who utilized adipose pad grafts in nerve surgery to mitigate the risk of postoperative neuromas and shield the nerve from external pressures [35]. The adipose pad provides a cushioning effect around the nerve, isolating it from surrounding tissues and reducing the risk of neuroma formation and pain [35, 36]. Several other studies could show that an increased amount of adipose tissue surrounding the transected nerve (e.g. by fat grafting) accelerates neuronal regeneration and prevents disorganized axonal outgrowth because of increased vascularization and reduced inflammatory processes, and secondary decreased fibrosis and hypertrophy of the connective tissues. Additionally, adipose tissue prevents strangling of the transected nerve by contraction of the surrounding tissues and entrapment [37–42]. Finally, adipose tissue has also recently been shown to play a paracrine role in promoting a metabolic shift in Schwann cells that is necessary for an appropriate repair response to injury [34].

Despite the localization of adipose tissue within rather than surrounding, the nerves in our study, its potential protective role for nerve fascicles cannot be discounted. The diminished cushioning from increased neuroma pressure could enhance spontaneous afferent signals to the spinal cord, potentially heightening sensitivity in nociceptive fibers and promoting both peripheral and central sensitization. Yet, our findings reveal no distinction in pain experience between patients with or without neuromas, indicating that the presence of adipose tissue within the neuroma does not directly influence pain perception.

In our study, we observed that the balance between organized and unorganized nervous tissue was closely associated with pain intensity. Patients reporting neuroma pain were noted to have a predominance of unorganized over organized nervous tissue, a disparity underscored by comparing the absolute areas covered by each tissue type. Remarkably, the area occupied by organized nervous tissue was substantially larger in patients without neuroma pain.

However, when correlating these morphological characteristics with pain levels, no significant relationship was found with the absolute nor relative measures of the identified criteria. Nonetheless, a significant negative correlation was observed between pain levels and the relative amount of organized nervous tissue, as well as the ratio of organized to unorganized tissue. This suggests a complex interplay between tissue organization within neuromas and the manifestation of pain, highlighting the intricate dynamics of neuropathic pain mechanisms.

A possible explanation for the observed association between preserved fascicular tissue and lower pain intensity may lie in principles of the Gate Control Theory of Pain [43]. Organized fascicles could help maintain a balanced input of nociceptive and non-nociceptive fibers to the spinal cord, whereas disorganized neuroma tissue, with its predominance of unmyelinated C- and thin Aδ-fibers [44–46] may shift this balance towards nociceptive signaling. This disproportion is further supported by the upregulation of neurotrophic factors after injury, which preferentially stimulate regeneration of unmyelinated fibers [10].

A translational implication of our findings is whether fascicular preservation can be assessed in vivo. High-resolution ultrasound (HRUS) and MR neurography (MRN) delineate neuroma morphology and continuity, but current implementations offer limited spatial resolution for intraneural fascicular architecture. In cadaveric histology correlation, 22-MHz HRUS identified ~ 51–74% of fascicles, compared with ~ 87–92% by magnetic resonance microscopy [47]. MRN depicts nerve course and signal change but cannot reliably resolve fascicular detail [48]. Diffusion-based techniques (DTI/tractography) may infer fiber orientation, though applications in neuromas remain investigational [49, 50]. Ultra-high-frequency ultrasound (~ 70 MHz) can visualize fascicular detail in superficial nerves and has been reported for traumatic neuroma and lipofibromatous hamartoma, but depth penetration is limited [51, 52]. Together, these modalities highlight the potential, yet current limits, of translating fascicular morphometry into non-invasive diagnostics.

To conclude, while most studies addressed the origins of aberrant nociceptive signaling, our results suggest that the degree of preserved internal nerve organization plays a critical role in modulating pain perception. This structural integrity may act as a buffer against the functional consequences of neuroma degeneration, offering a new morphometric biomarker for predicting pain severity.

Importantly, while our approach captures static structural features, it does not account for dynamic neural signaling or central sensitization, which most probably also contribute to pain variability. In addition, our study is limited by its focus on a single region of transversely cut nerves, leaving open the question of how other regions might contribute to inter sample variability, and by its reliance on purely morphological rather than molecular characterization. Finally, one limitation of our study is incomplete clinical metadata: duration of injury and exact nerve function (motor, sensory, or mixed) could not be consistently retrieved from retrospective charts. Nevertheless, all lesions represent Sunderland grade V injuries, as they resulted from complete transections in amputation or tumor resection. However, the observed associations between structural integrity and pain perception remain robust.

## Conclusion

This study provides the first quantitative evidence that the internal structural organization of neuromas, specifically the relative preservation of healthy nerve fascicles, is a critical determinant of pain. This contributes to a growing understanding of neuroma-associated pain by highlighting the potential relevance of internal nerve organization. While previous work has focused on identifying the sources of aberrant nociceptive firing, ranging from immune infiltration to fibrotic remodeling, our findings indicate that such signals may be modulated, buffered, or counteracted by the amount of intact, functional neural tissue. This challenges the notion that neuroma pain is determined solely by what triggers it and emphasizes instead how well the nerve retains its internal order. These insights support a shift from solely etiological models of neuroma pain toward structurally-informed diagnostics and therapeutic strategies. Overall, these mechanisms are likely complementary rather than exclusive, and further research is needed to determine how they interact in modulating neuropathic pain.

## Supplementary Information

Below is the link to the electronic supplementary material.

Supplementary Material 1

## Data Availability

Data is provided within the manuscript or supplementary information files.
