# Correction: Preserved fascicular architecture predicts neuroma pain: a morphometric study

**DOI:** 10.1186/s40478-026-02239-5

**Published:** 2026-02-17

**Authors:** Luis A. Pardo, Carolina Thomas, Arndt F. Schilling, Christine Stadelmann, Jennifer Ernst

**Affiliations:** 1 Hannover Medical School, Department of Trauma Surgery, Hannover, Germany; 2 Department of Trauma Surgery, Orthopaedics and Plastic Surgery, University Medical Center Göttingen, Göttingen, Germany; 3 Department of Neuropathology, University Medical Center Göttingen, Göttingen, Germany; 4 Paul Flechsig Institute – Centre of Neuropathology and Brain Research, Medical Faculty, Leipzig University, Leipzig, Germany

**Correction to: Acta Neuropathologica Communications (2025) 13:248** 10.1186/s40478-025-02154-1

In this article [1], the affiliations 1 and 2 were wrongly numbered; Affiliation 1 should have been affiliation 2 and vice versa as shown below.

Incorrect affiliation order:

1. Department of Trauma Surgery, Orthopaedics and Plastic Surgery, University Medical Center Göttingen, Göttingen, Germany

2. Department of Trauma Surgery, Hannover Medical School, Hannover, Germany

Correct affiliation order:

1. Hannover Medical School, Department of Trauma Surgery, Hannover, Germany

2. Department of Trauma Surgery, Orthopaedics and Plastic Surgery, University Medical Center Göttingen, Göttingen, Germany

And, the e-mail address of the corresponding author Luis A. Pardo was incorrectly given as luis.pardo@med.uni-goettingen.de where it should have been PardoSanchez.Luis@mh-hannover.de.

The original article has been corrected.
