# Supplementary material for: Preserved fascicular architecture predicts neuroma pain: a morphometric study
Source: Acta Neuropathol Commun. 2025 Dec 1;13:248. doi: 10.1186/s40478-025-02154-1 (PMC12670820; doi:10.1186/s40478-025-02154-1)
Supplement: Supplementary file 1 — Supplementary Material 1 [file 40478_2025_2154_MOESM1_ESM.docx]

**Supplementary Materials**

**
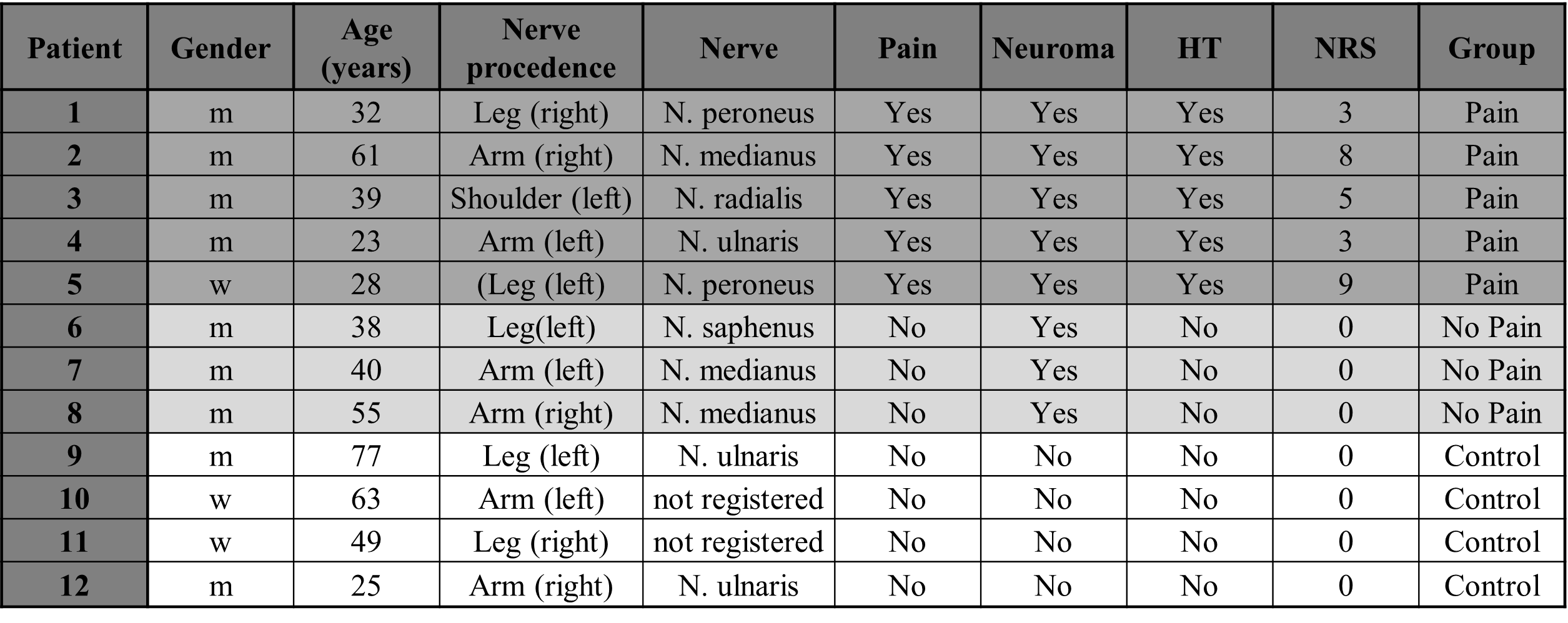
**

**Table 1.: Tissue samples.** Patients with neuroma pain underwent surgery. Patients with neuroma but without pain underwent surgery for refashioning of the residual limb. If a neuroma was detected intraoperatively it was treated. The control group are transected nerves, without pain or neuroma. These were extracted during tumor surgery. The pain level was obtained from the patient using a numeric rating scale (NRS), the Hoffmann-Tinel sign (HT) was elicited by proximally tapping on the affected nerve.

EvG whole slide images were analyzed using a random forest classifier from scikit-learn [38], in which a pixel-based segmentation was computed using local, morphology features based on geometry as local intensity, edges and textures. In a first step, tissue components were annotated using several regions of interest (ROI) per image.

Thus, following categories were included: “background”, “organized nervous tissue”, “unorganized nervous tissue”, “connective tissue”, “fat”, and “erythrocytes” (Figure 1). These ROIs were extracted and transformed into a 1-dimensional mask, in which each pixel was either 0 (unlabeled), or a number between 1 and 6 respectively for every tissue component. The pixels of the mask were used to train a random-forest classifier. For computational reasons, the images and ROI-masks were split into four equal pieces, trained separated, and stitched together afterwards. In the next step, the classifier predicted the unlabeled pixels and output a 6-dimensional matrix (one dimension per tissue category). The mask containing healthy fascicles was further filtered in an additional step, as the algorithm annotated healthy fascicles as neuromas in some cases. Here, we used the inherent roundness of healthy fascicles to exclude mislabeled pixels. First, utilizing a Laplacian transform we defined the borders of the masks. To enhance the segmentation of single fascicles, we initially expanded the boundaries of the mask through dilation and then subtracted these dilated borders from the original mask. Given that fascicles typically exhibit a circular geometry, we adopted the premise that circular structures correspond to fascicles (organized nervous tissue). Consequently, we proceeded to identify the largest circle that could be accommodated within each fascicle, as well as the smallest circle that encompassed the entire fascicle. This approach allowed us to introduce a criterion based on the radial ratio, the quotient of the radii of these two circles, to assess fascicle health. Specifically, we stipulated that a fascicle could be considered "healthy" if the radial ratio did not exceed 0.2. Fascicles failing to meet this criterion were not included in the "healthy fascicle" category. Instead, these circular entities were classified as "mini fascicles," a distinction that facilitated the nuanced analysis of fascicle integrity and morphology.

The masks were then used to define the percentage of each tissue in relation to the whole nerve, the ratio between healthy fascicle and neuroma, as well as the amount of connective tissue inside the neuromas. Using the percentual amount of white pixels in relation to the black ones in previously mentioned masks, we calculated the relative area of organized nervous tissue, unorganized nervous tissue, connective tissue, fat, and erythrocytes. Additionally, we calculated the relative area of connective tissue intruding neuroma by creating a new mask surrounding the interconnected neuroma by performing a convex hull, and binary multiplying this new mask with the connective tissue mask. This results in a mask only containing the connective tissue which is located in the surroundings of unorganized fascicles. Absolute area for a tissue category were determined by multiplying its known percentage by the absolute image area.

The ratio between the amount of organized and unorganized fascicles was calculated when using the following formula $\frac{unorganized-organized}{max(unorganized, organized)}$. This results in a value between -1 and 1, from which 1 means, that only unorganized nervous tissue is present and -1, that only organized nervous tissue is present (which is only true for the control group).

We used the resulting values to correlate relative and absolute amounts with the pain level reported by patients with neuroma, obtained from the medical records collected by a physician using a visual scale (VAS).

| **Comparison** | **Variable 1** | **Variable 2** | **Results 1** | **Results 2** | **p-value** |
| --- | --- | --- | --- | --- | --- |
| Neuroma (%) | controls | patients | 0.0 [0.0] | 35.5 [32.8] | 0.007 |
| Fascicles (%) | controls | patients | 33.5 [16.0] | 6.2 [18.8] | 0.106 |
| Connective tissue (%) | controls | patients | 30.8 [12.9] | 31.5 [15.5] | 0.515 |
| Adipose tissue (%) | controls | patients | 25.7 [19.0] | 6.9 [8.0] | 0.030 |
| Neuroma (mm²) | controls | patients | 0.0 [0.0] | 20.2 [74.9] | 0.007 |
| Fascicles (mm²) | controls | patients | 4.6 [7.4] | 9.0 [10.5] | 0.799 |
| Connective tissue (mm²) | controls | patients | 3.9 [3.6] | 28.1 [38.8] | 0.066 |
| Adipose tissue (mm²) | controls | patients | 2.5 [11.4] | 5.3 [5.4] | 0.562 |
| Nerve size (mm²) | controls | patients | 11.1 [24.7] | 86.7 [99.7] | 0.135 |
| Neuroma (%) | no pain | pain | 16.5 [11.3] | 36.9 [24.9] | 0.113 |
| Fascicles (%) | no pain | pain | 37.4 [17.3] | 3.7 [4.1] | 0.006 |
| Connective tissue (%) | no pain | pain | 26.1 [7.0] | 39.1 [21.6] | 0.618 |
| Adipose tissue (%) | no pain | pain | 1.6 [10.4] | 8.6 [3.8] | 0.623 |
| Intruding conn. tissue (%) | no pain | pain | 1.9 [6.5] | 15.5 [6.6] | 0.172 |
| Neuroma incl. conn. tissue (%) | no pain | pain | 25.9 [14.4] | 58.9 [34.2] | 0.150 |
| Neuroma (mm²) | no pain | pain | 9.3 [12.8] | 68.2 [88.0] | 0.232 |
| Fascicles (mm²) | no pain | pain | 14.3 [11.1] | 5.5 [11.5] | 0.162 |
| Connective tissue (mm²) | no pain | pain | 14.0 [14.7] | 68.1 [55.3] | 0.219 |
| Adipose tissue (mm²) | no pain | pain | 0.9 [1.8] | 7.8 [2.2] | 0.147 |
| Intruding conn. tissue (mm²) | no pain | pain | 0.6 [6.0] | 10.3 [20.5] | 0.283 |
| Neuroma incl. conn. tissue (mm²) | no pain | pain | 14.6 [20.9] | 104.3 [96.9] | 0.263 |
| Nerve size (mm²) | no pain | pain | 56.3 [35.1] | 131.9 [103.8] | 0.232 |
| Normalized deviation | no pain | pain | -0.7 [0.6] | 0.9 [0.1] | 0.006 |

**Table 2 - Morphological analysis of controls and neuromas**

**Figure design and further software**

All figures in this manuscript, including graphical representations were created with BioRender.com or Microsoft PowerPoint. All plots showing experimental results were created using Seaborn [24] and statistical annotation were included using Statannotations [25]; both libraries are available for Python. For grammar and style, Grammarly.com suggestions were taken into account.
